# Environmental Factors Affect the Bacterial Community in *Diaphorina citri*, an Important Vector of “*Candidatus* Liberibacter asiaticus”

**DOI:** 10.1128/spectrum.05298-22

**Published:** 2023-03-28

**Authors:** Rui-Xu Jiang, Feng Shang, Hong-Bo Jiang, Wei Dou, Tomislav Cernava, Jin-Jun Wang

**Affiliations:** a Key Laboratory of Entomology and Pest Control Engineering, College of Plant Protection, Southwest University, Chongqing, China; b International Joint Laboratory of China-Belgium on Sustainable Crop Pest Control, Academy of Agricultural Sciences, Southwest University, Chongqing, China; c Institute of Environmental Biotechnology, Graz University of Technology, Graz, Austria; Fujian Agriculture and Forestry University

**Keywords:** Asian citrus psyllid, bacterial community, environmental factors, *Wolbachia*, citrus Huanglongbing

## Abstract

Insects are associated with diverse microbial communities that can have substantial effects on hosts. Here, we characterized the bacterial communities in the Asian citrus psyllid (ACP), Diaphorina citri (Hemiptera: Psyllidae), a major vector of the devastating pathogen “*Candidatus* Liberibacter asiaticus,” which causes citrus Huanglongbing (HLB). In total, 256 ACP individuals across 15 field sites and one laboratory population in China were sequenced. The results showed that the bacterial community diversity was the highest in the Guilin population (average Shannon index, 1.27), and the highest value for richness was found in the Chenzhou population (average Chao1 index, 298). The bacterial community structures of the field-collected populations were significantly different, and all of them harbored *Wolbachia*, which was assigned to strain ST-173. Structural equation models revealed that the dominant *Wolbachia* strain had a significantly negative correlation with the annual mean temperature. In addition, the results obtained with populations infected with “*Ca.* Liberibacter asiaticus” indicated that in total, 140 bacteria could be involved in interactions with this bacterium. The ACP field populations harbored a more diverse bacterial community than the laboratory population, and the relative occurrences of some symbionts differed significantly. However, the bacterial community of the ACP laboratory colony was connected in a more complex network structure (average degree, 54.83) than that of the field populations (average degree, 10.62). Our results provide evidence that environmental factors can influence the bacterial community structure and bacterial relative abundance in ACP populations. This is likely due to the adaptation of ACPs to local environments.

**IMPORTANCE** The Asian citrus psyllid (ACP) is an important vector of the HLB pathogen, which is a major threat to citrus production around the world. Bacterial communities harbored by insects could be affected by different environmental factors. Understanding these factors that affect the bacterial community of the ACP could be important for the better management of HLB transmission. This work surveyed ACP field populations in mainland China in order to explore the bacterial community diversity of different populations and the potential relationships between environmental factors and predominant symbionts. We have assessed the differences in ACP bacterial communities and identified the prevalent *Wolbachia* strains in the field. In addition, we compared the bacterial communities of ACP field-collected and laboratory populations. Comparing populations subjected to contrasting conditions could help us to better understand how the ACP adapts to local environmental conditions. Our study provides new insights into how environmental factors influence the bacterial community of the ACP.

## INTRODUCTION

Endosymbionts have various important roles in insect physiology (1, 2), including the regulation of the host’s development (3) and fecundity (4) as well as the provision of essential amino acids to the host (5, 6). Alternatively, various factors can affect the diversity and proportion of insect symbionts within the entire microbial community that colonizes them (7). For example, the occurrence of *Spiroplasma* can reduce the density of *Wolbachia* in *Drosophila* (8), and *Asaia* prevents the vertical transmission of *Wolbachia* in Anopheles stephensi (9). Previous studies showed that the same species of insects can harbor endosymbiont populations of substantially different diversities in natural populations (10). Therefore, it is assumed that the endosymbiont and overall microbial community diversity could be affected by specific factors, including the host species (11, 12), local feed resources (13), pH (14), as well as the annual mean temperature and precipitation (15, 16). It was previously reported that the Asian citrus psyllid (ACP) harbors different endosymbionts (17, 18) and that different strains of *Wolbachia* can exist in ACPs (19). However, it is still unclear how environmental factors affect bacterial communities associated with the ACP and which *Wolbachia* strains are dominant in different ACP populations in China.

The ACP, Diaphorina citri Kuwayama (Hemiptera: Psyllidae), is an insect that feeds on phloem and is an important vector of “*Ca*. Liberibacter asiaticus,” more commonly known as the citrus Huanglongbing (HLB) pathogen, which causes serious damage to citrus production around the world (20). The ACP is relatively ineffective at migrating (21, 22), which renders it a suitable model to investigate the impact of environmental factors on insect endosymbionts.

In this study, we surveyed field populations of ACPs in the primary regions of the Chinese mainland that produce citrus. The main objectives of this study were to assess differences in the ACP bacterial communities and to identify the prevalent *Wolbachia* strains in the field. In addition, we compared the bacterial communities of ACP field-collected and laboratory populations. We hypothesized that variable environmental conditions would result in substantially different bacterial community compositions, in contrast to laboratory conditions, which are continuously stable. These contrasting conditions could help us to better understand the importance of environmental factors that play a role in shaping the bacterial communities associated with ACPs. Therefore, we utilized 16S rRNA gene fragment amplicon sequencing to investigate the diversity and composition of bacterial communities at the population level. Our findings will enhance currently existing knowledge related to bacterial associations with insects.

## RESULTS

### Complementary identification of *Wolbachia* strains in the ACP populations.

**FIG 1:**
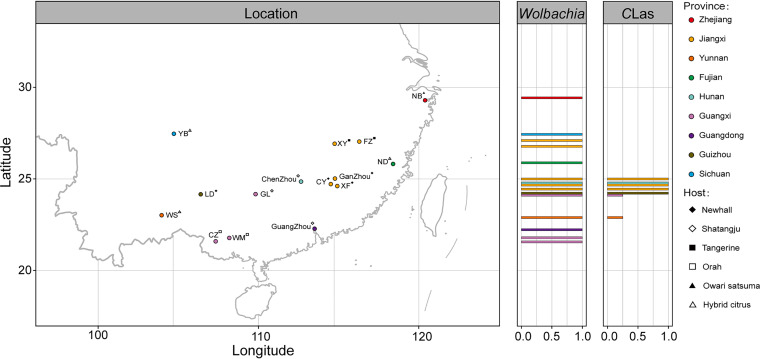
(Left) Sampling sites for field populations of the Asian citrus psyllids (ACP) across 15 major citrus-producing regions in China. (Right) Occurrence of *Wolbachia* or “*Candidatus* Liberibacter asiaticus” (*C*Las) in field populations. Positions correspond to the latitude of the field population.

**FIG 2:**
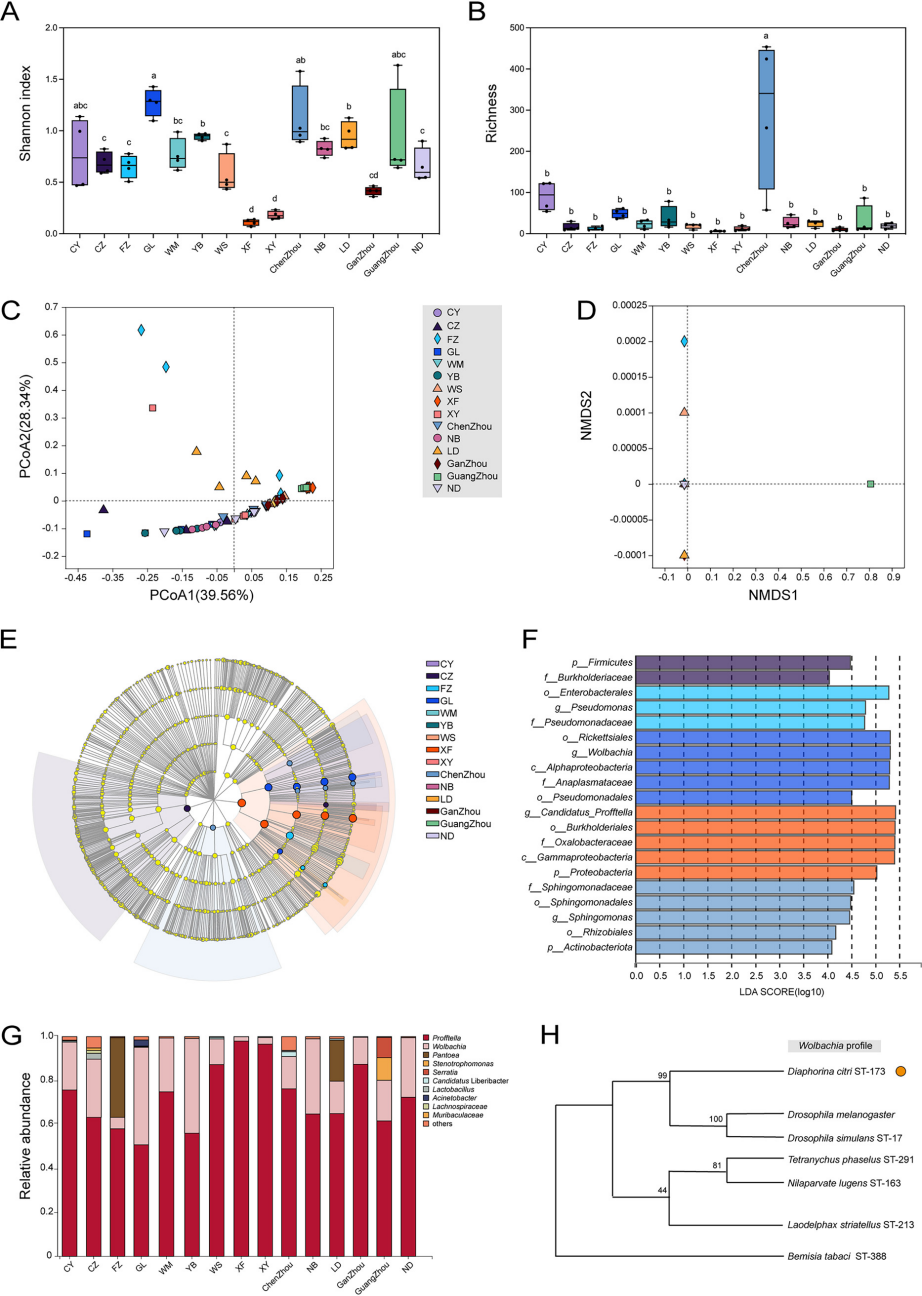
Bacterial communities in different field populations of the Asian citrus psyllid (ACP). (A and B) α-Diversities, including Shannon and richness (Chao1) indices, of the bacterial communities in different ACP populations. Different letters represent significant differences between field ACP groups at the level of a *P* value of <0.05 based on the Newman-Keuls test. (C and D) Principal-coordinate analysis (PCoA) and nonmetric multidimensional scaling (NMDS) of bacterial communities based on Bray-Curtis dissimilarities. The variation explained by the PCoA and NMDS axes is provided in parentheses. (E) Cladogram representation. (F) Predominant bacteria of ACP populations with an LDA score of >4. Labels beginning with “o” indicate order, “f” indicates family, “g” indicates genus, and “s” indicates species. (G) Relative abundances of bacterial taxa at the genus level from 15 field ACP populations. (H) Bayesian analysis of five genes of *Wolbachia* from 15 wild ACP populations with different *Wolbachia* strains.

All of the field-collected ACP samples were shown to be infected with *Wolbachia* (Fig. 1). The five allele sequences of all of the individuals that were sampled in the field in this study completely matched those of the strain belonging to sequence type 173 (ST-173) (Table 1) in the MLST (multilocus sequence typing) database. According to the available database entries, the identified strain is closely related to Drosophila melanogaster ST-13 and Drosophila simulans ST-17 (Fig. 2H).

**TABLE 1:** *w*Di allelic profiles identified in 15 ACP populations^*a*^

Location	Allele					Strain type
	*gatB*	*hcpA*	*coxA*	*ftsZ*	*fbpA*	
Ganzhou	109	29	86	81	27	ST-173
Chongyi	109	29	86	81	27	ST-173
XF	109	29	86	81	27	ST-173
XY	109	29	86	81	27	ST-173
FZ	109	29	86	81	27	ST-173
GL	109	29	86	81	27	ST-173
CZ	109	29	86	81	27	ST-173
WM	109	29	86	81	27	ST-173
NB	109	29	86	81	27	ST-173
ND	109	29	86	81	27	ST-173
LD	109	29	86	81	27	ST-173
WS	109	29	86	81	27	ST-173
Chenzhou	109	29	86	81	27	ST-173
Guangzhou	109	29	86	81	27	ST-173
YB	109	29	86	81	27	ST-173

a ACP, Asian citrus psyllid; CY, Chongyi; XF, Xinfeng; XY, Xinyu; FZ, Fuzhou; GL, Guilin; CZ, Chongzuo; WM, Wuming; NB, Ningbo; ND, Ningde; LD, Luodian; WS, Wenshan; YB, Yibin.

### Bacterial community diversity in different ACP field populations.

The α-diversity indices were estimated using the Shannon index and bacterial richness (Chao1 index) across the 15 field populations of ACPs. The results indicated that the GL (Guilin) population had the highest Shannon index value (1.27) of all of the field populations, while the XF (Xinfeng) population had the lowest value (0.11) (see Fig. 2A). The Chenzhou population had the highest Chao1 index value (298.2) compared with the other field populations (see Fig. 2B; see also Table S3 in the supplemental material). Based on Bray-Curtis dissimilarity, significant variations the in bacterial compositions of the field populations were confirmed (*R*^2^ = 0.5640; *P* = 0.001 [by ADONIS]) and plotted with a principal-coordinate analysis (PCoA), with PCoA1 (39.56%) and PCoA2 (28.34%) explaining 67.9% of the variation (see Fig. 2C). Complementary analyses were conducted by nonmetric multidimensional scaling (NMDS) (*R*^2^ = 0.5640; *P *= 0.001 [by ADONIS]) (see Fig. 2D). All of the field populations exhibited a high relative abundance of *Proteobacteria*. However, the CZ (Chongzuo), CY (Chongyi), and Chenzhou populations were also associated with *Firmicutes* (Fig. S1). Taxonomic classification with the RDP classifier identified 2,465 amplicon sequence variants (ASVs) that belonged to 892 species, 603 genera, and 322 families. When the overlap of endosymbionts among the different ACP populations was assessed, only three ASVs were shared by all of the samples (Fig. S2). In addition, the linear discriminant analysis (LDA) effect size (LEfSe) algorithm was used to identify significantly enriched taxa within the different field populations. A total of 603 taxa were found to have significant differences in their relative abundances. Detailed assessments showed that *Wolbachia* was enriched in the GL population, Pseudomonas had the highest relative abundance in the FZ (Fuzhou) population, *Ca*. Profftella had the highest relative abundance in the XF population, and *Sphingomonas* had the highest relative abundance in the Chenzhou population (see Fig. 2E and F). Moreover, the endosymbionts *Ca*. Profftella and *Wolbachia* were enriched at different ranks in all of the ACP populations (see Fig. 2G and Fig. S3A). *Ca*. Profftella accounted for 97.8% of the bacterial community in the XF population. This was the highest relative abundance of this bacterium within all of the populations. In contrast, the GL population harbored the lowest proportion of *Ca*. Profftella (52.52%). As the primary endosymbiont, *Wolbachia* accounted for the highest proportion (43.51%) in the GL population and the lowest proportion (2.09%) in the XF population. Other prominent genera, including *Pantoea* and *Ralstonia*, occurred at different relative abundances in the ACP field populations (Fig. S3B).

### Identification of environmental factors that correlate with microbial community members in ACPs.

**FIG 3:**
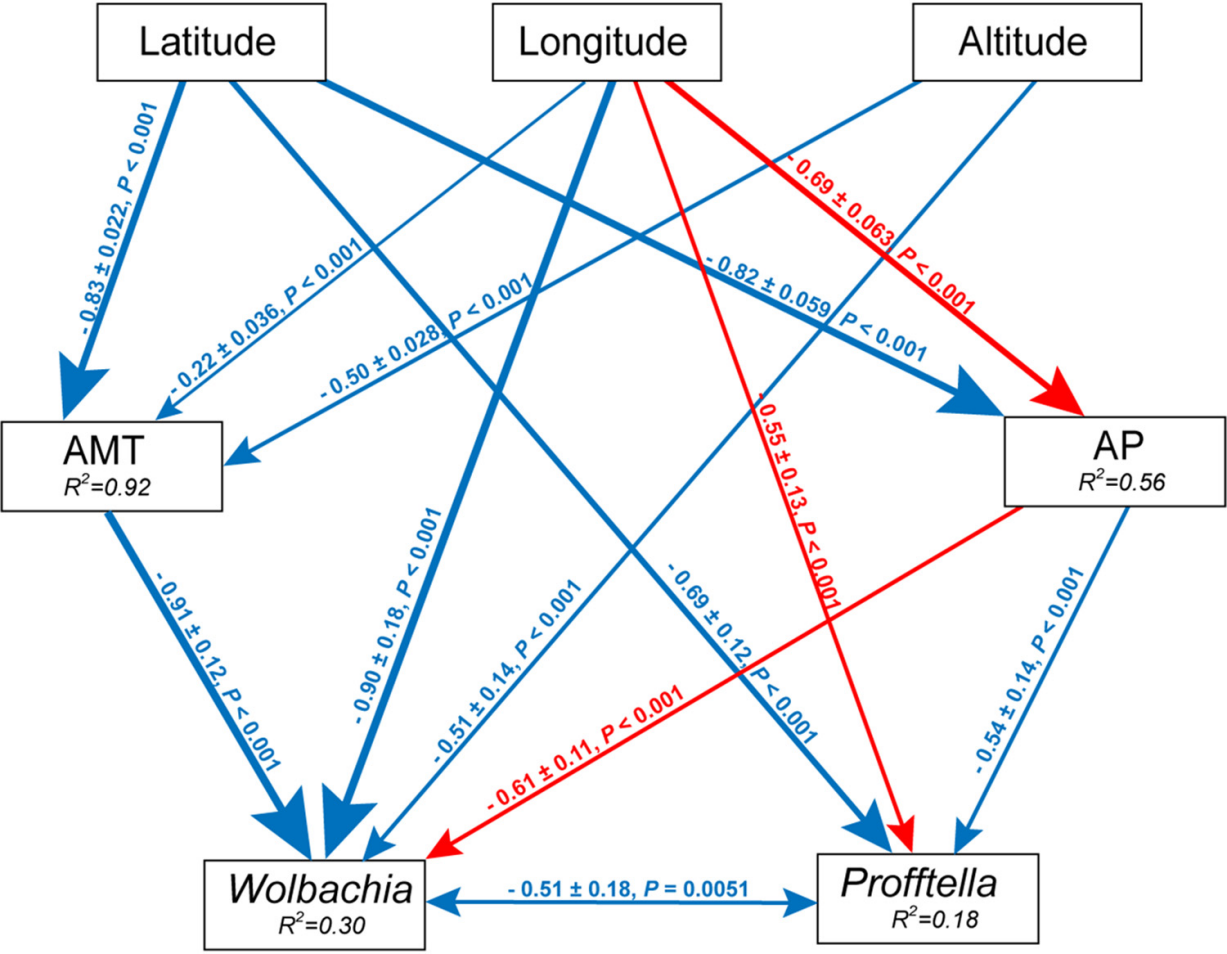
Effects of various factors on the bacterial community compositions of different field populations of the Asian citrus psyllid (ACP). The path diagram is based on the structural equation model (SEM) for environmental factors and microbial Bray-Curtis dissimilarity. The red solid and blue solid lines represent significant positive and negative paths, respectively. The arrow widths represent the strengths of these relationships. The *R*^2^ values of every box indicate the amount of variation in that variable explained by the input arrows. The numbers next to the arrows are unstandardized slopes.

The results showed that *Ca*. Profftella, *Wolbachia*, *Lactobacillus*, and Acinetobacter were significantly spatially autocorrelated. In contrast, *Pantoea* and “*Ca.* Liberibacter asiaticus” showed no significant spatial autocorrelation. In addition, the annual mean temperature and annual precipitation were significantly spatially autocorrelated (Table S4). A structural equation model (SEM) was used to explore the relationships between ACP symbionts and five putative predictive variables (altitude, average mean temperature [AMT], average precipitation [AP], latitude, and longitude). Based on the exclusion of 11 paths (χ^2^ = 5.06, df = 18, *P *= 0.28, comparative fit index [CFI] = 0.998, Akaike information criterion [AIC] value = 704.44, and standardized root mean square residual [SRMR] = 0.039), the SEM was accepted. The results showed that AMT significantly decreased with latitude (−0.83 ± 0.022; *z* = 38.13; *P < *0.001), longitude (−0.22 ± 0.036; *z* = 6.07; *P < *0.001), and altitude (−0.50 ± 0.028; *z* = 17.79; *P < *0.001). AP significantly decreased with latitude (−0.82 ± 0.059; *z* = 14.05; *P < *0.001) and increased with longitude (0.69 ± 0.063; *z* = 10.93; *P < *0.001). The proportion of *Wolbachia* significantly decreased with AMT (−0.91 ± 0.12; *z* = 7.63; *P < *0.001), longitude (−0.90 ± 0.18; *z* = 5.14; *P < *0.001), and altitude (−0.51 ± 0.14; *z* = 3.60; *P < *0.001) and increased with AP (0.61 ± 0.11; *z* = 5.57; *P < *0.001). The proportion of *Ca*. Profftella significantly decreased with latitude (−0.69 ± 0.063; *z* = 10.93; *P < *0.001) and increased with longitude (0.55 ± 0.13; *z* = 4.29; *P < *0.001). The ACP symbiont *Wolbachia* was negatively correlated (−0.51 ± 0.18; *z* = 2.78; *P *= 0.0051) with *Ca*. Profftella (Fig. 3). The proportion of *Wolbachia* (49.96%) was influenced by the indirect effects of climate factors as a function of spatial factors (Table S5).

### Relationships between “*Ca.* Liberibacter asiaticus” and different ACP endosymbionts.

**FIG 4:**
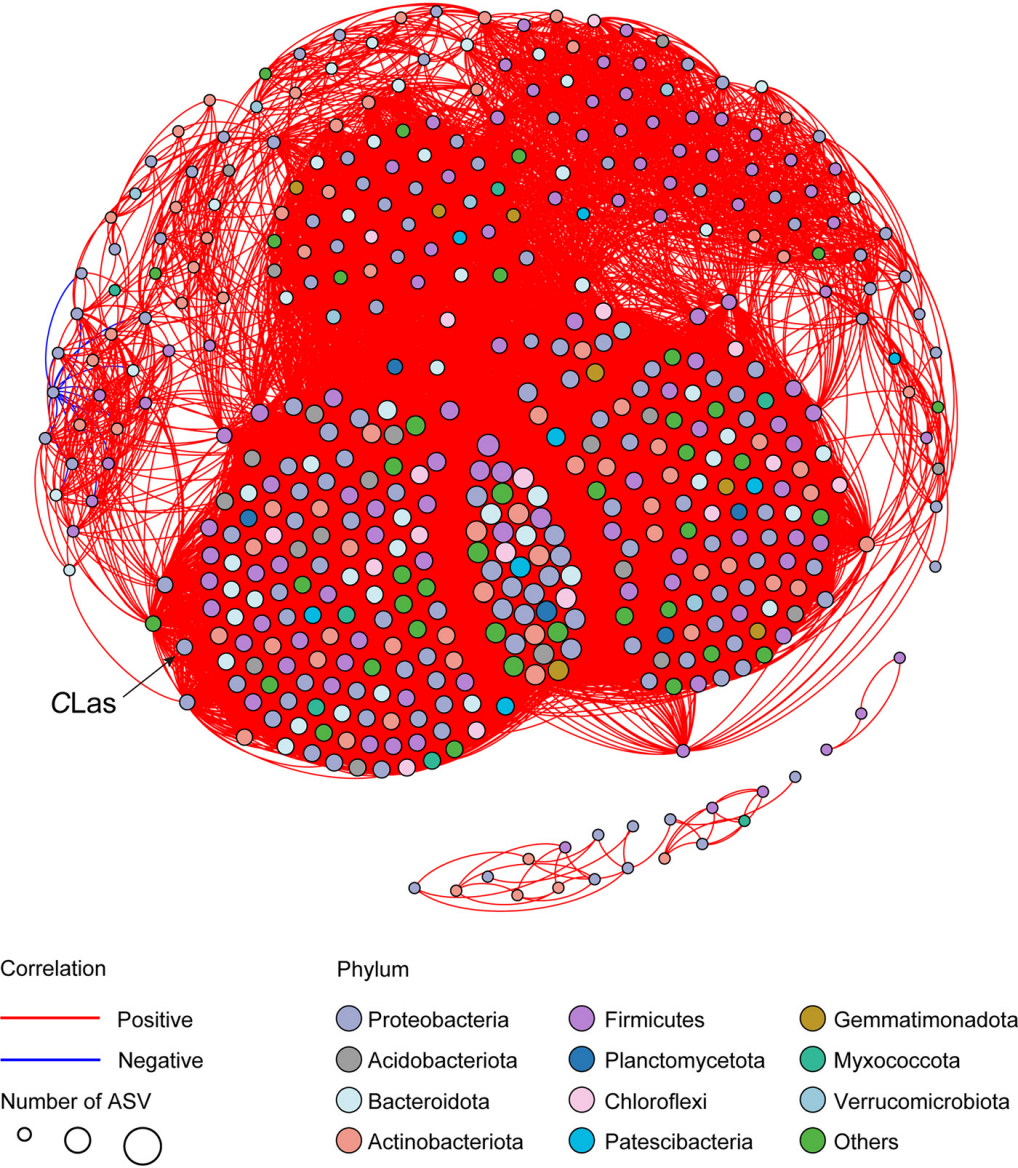
Networks of the bacterial communities detected in field populations of the Asian citrus psyllid (ACP) infected with “*Candidatus* Liberibacter asiaticus” (*C*Las). Edges represent significant Spearman correlations (ρ > |0.5|; *P < *0.05). The red and blue lines represent significant positive and negative correlations, respectively. The sizes of the circles indicate the numbers of amplicon sequence variants (ASVs).

“*Ca.* Liberibacter asiaticus” was detected in seven ACP populations collected from the field (Fig. 1). The 16S rRNA gene fragment amplicon subset from the “*Ca.* Liberibacter asiaticus”-infected populations was subjected to NetworkX analysis to identify bacterial community members that potentially interact with the pathogen. The cooccurrence patterns between “*Ca.* Liberibacter asiaticus” and ACP symbionts were assessed with a Spearman correlation (ρ) cutoff value of >0.5 (Fig. 4). The uncultured genus Ellin6067 and *Idiomarina* showed the highest correlations with “*Ca.* Liberibacter asiaticus.” In total, 140 strains were found to have a positive association with “*Ca.* Liberibacter asiaticus” (Fig. S4 and Table S6).

### Comparison of bacterial communities in the ACP field-collected and laboratory populations.

**FIG 5:**
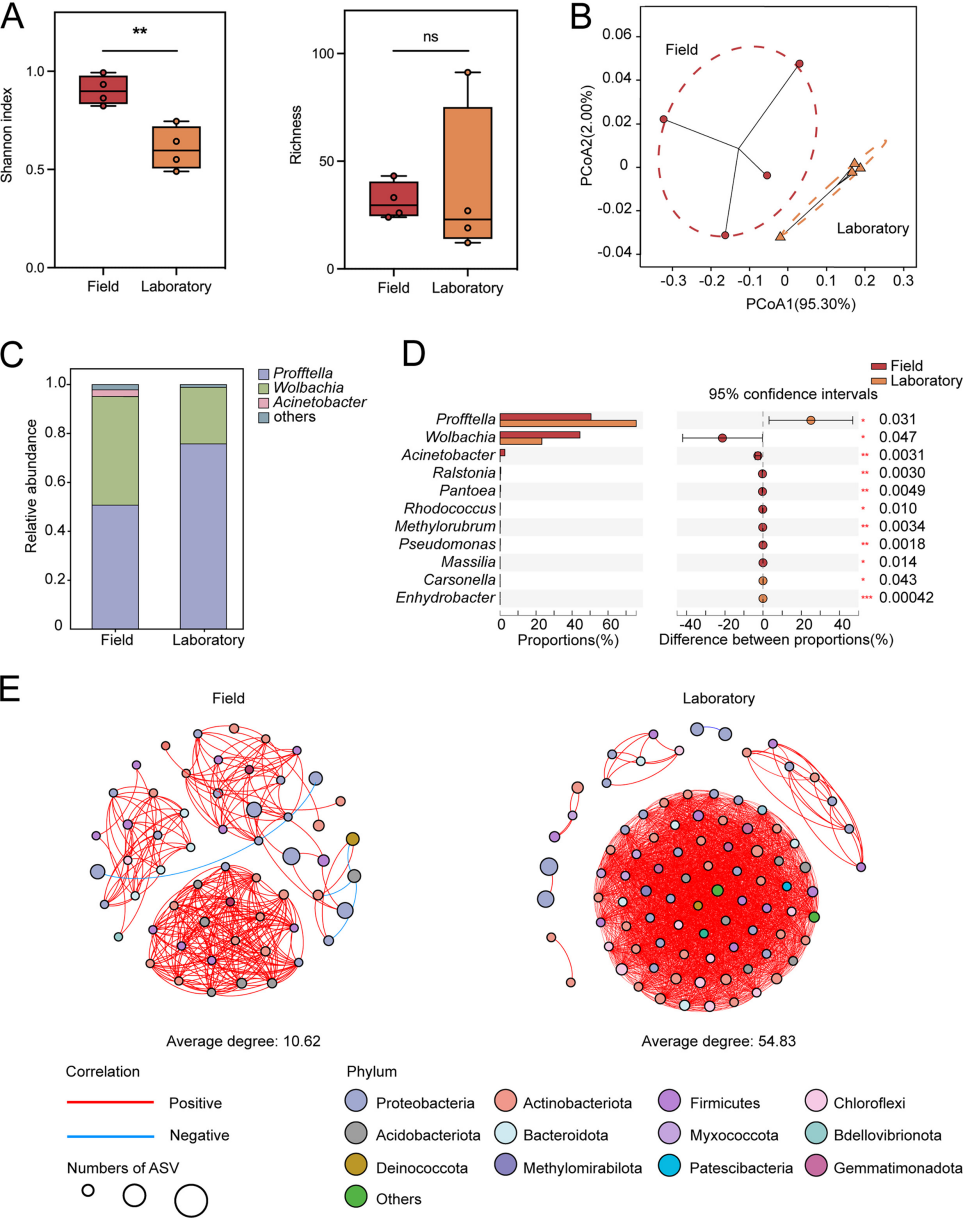
Diversity and community structure of bacterial communities in Asian citrus psyllid (ACP) field and laboratory populations. (A) Shannon and richness (Chao1) indices of the bacterial communities in the field and laboratory ACP populations. The asterisk represented significant differences between groups at the level of a *P* value of <0.05 based on Student’s *t* test (ns, not significant; **, *P < *0.01). (B) Principal-coordinate analysis (PCoA) of bacterial community Bray-Curtis dissimilarities. (C) Relative abundances of bacterial taxa at the genus level in field and laboratory populations. (D) Comparison of the proportions of specific bacterial genera associated with ACPs between field and laboratory populations. Different letters represent significant differences between groups at the level of a *P* value of <0.05 based on Student’s *t* test (ns, not significant; *, *P < *0.05; **, *P < *0.01; ***, *P < *0.001). (E) Networks of the bacterial communities found in ACP field and laboratory populations. Edges represent significant Spearman correlations (ρ > |0.6|; *P* < 0.05). The red and blue lines represent significant positive and negative correlations, respectively. The circle sizes indicate the numbers of amplicon sequence variants (ASVs).

We compared the bacterial communities between ACP field-collected and laboratory populations without considering the effects of environmental factors. When their α-diversity values were compared, the Shannon index of bacterial communities in the ACP field-collected populations was significantly higher (*P *= 0.00062 [by Student’s *t* test]) than that of the communities in the laboratory population, but the richness (Chao1 index) values were not significantly different between the ACP field-collected and laboratory populations (Fig. 5A). An assessment of the β-diversity indicated that the ACP field-collected and laboratory populations had similar bacterial compositions (*R*^2^ = 0.5546; *P* = 0.071 [by ADONIS]) (Fig. 5B and C). However, the proportions of bacteria occurring in the ACP populations were significantly different. For example, *Ca*. Profftella had a higher proportion in the ACP laboratory population than in the field-collected populations (*P *= 0.031 [by Student’s *t* test]) (Fig. 5D). The network complexity of the laboratory population (average degree, 54.83) was higher than that of the field-collected populations (average degree, 10.62) (Fig. 5E). The number of edges was higher in the laboratory population (2,521 positive edges and 1 negative edge) than in the field-collected populations (304 positive and 4 negative edges) (Table S7).

## DISCUSSION

To our knowledge, this study is the first to investigate the bacterial communities associated with ACPs at the population level. The overall results showed that the ACP field populations differed in their bacterial community diversity, which could be due to differences in environmental factors. Especially, factors such as AMT, longitude, and latitude affected the composition of ACP bacterial communities. Some of these factors could have promoted the ability of ACPs to adapt to local biodiversity, as previously described for other insects (23). In addition, the heritable endosymbionts of ACPs could have potentially further shaped the structure of the environmentally acquired bacterial communities. It was shown previously in a similar context that *Wolbachia* can alter microbial communities in wild populations of small brown planthoppers (Laodelphax striatellus) (24). Analogous results were also found for Egyptian mosquitoes (Aedes aegypti) (25), pill bugs (Armadillidium vulgare) (26), and beetles (27).

*Wolbachia* can modulate the insect host’s resource competition, immune system, and changes in metabolism (24, 25, 28). Various *Wolbachia* strains have been described to date, and they are known to have different functions (29–31). *Wolbachia* strains ST-173, Co-1, ST-FL, and Co-2 have been found in ACP populations around the world (19). In our study, we found that *Wolbachia* strain ST-173 was the dominant strain in ACP field-collected populations in the major citrus-producing regions in China.

In this study, the devastating pathogen “*Ca.* Liberibacter asiaticus” was detected in seven ACP field populations. “*Ca.* Liberibacter asiaticus” can be taken up by an ACP during feeding on host plant sap and spreads throughout its whole body, making the insect a vector for infections of healthy citrus plants (32). By conducting a cooccurrence analysis, we detected potential candidate bacteria that interact with “*Ca.* Liberibacter asiaticus.” Different media were used in efforts to isolate them; however, we were not successful in obtaining the target species (see Fig. S5 and S6 in the supplemental material). It was assumed in a previous study that *Wolbachia* could prevent “*Ca.* Liberibacter asiaticus” infection of the ACP (33). This could be specifically explored in upcoming studies by decreasing the titer of *Wolbachia* in the ACP to explore its specific association with “*Ca.* Liberibacter asiaticus.”

In the frame of this study, we also demonstrated that the bacterial communities associated with ACP field populations were more diverse than laboratory-reared populations. The same results were reported previously for other insects, including the fruit fly (*Drosophila*) (34) and the kissing bug (Triatoma infestans) (35). In addition, we observed that the microbial community of laboratory-reared ACPs had a better connection and a more complex network structure than those of field populations. This could be due to the variability in the degree of selection of the insect host or differences in external factors, such as laboratory versus field conditions, that affect the bacterial community (34).

In conclusion, our results provide a comprehensive overview of the bacterial community diversity and composition associated with the ACP. We highlight that environmental factors have substantial potential to shape the bacterial community in the ACP. Moreover, our results highlight specific environmental factors as important modulators that affect the bacterial community in insect species and provide a new vision of the shift in bacteria within insects among the continuum associated with geography and climate. However, we still do not know exactly the relationship between the ACP’s bacterial community and different plant host species. Future research will be required to explore how different plant species affect the ACP’s bacterial community and to understand the function of ACP-associated microorganisms and their adaption as well as their influence on the host’s adaptability to different ecosystems. A combination of different environmental factors could be implemented in the future to better predict ACP outbreaks and reduce fruit losses in citrus production.

## MATERIALS AND METHODS

### Sample collection and storage.

Samples of adult ACP individuals were collected from six different host plant species across 15 different citrus planting areas (Ningbo in Zhejiang Province; Ningde in Fujian Province; Fuzhou, Xinyu, Ganzhou, Chongyi, and Xinfeng in Jiangxi Province; Guangzhou in Guangdong Province; Chenzhou in Hunan Province; Guilin, Wuming, and Chongzuo in Guangxi Province; Luodian in Guizhou Province; Wenshan in Yunnan Province; and Yibin in Sichuan Province) that cover the primary citrus-producing regions in China (Fig. 1; see also Table S1 in the supplemental material). Sampling was conducted during the spring and summer (March to September) of 2021. Meteorological data, including altitude, latitude, longitude, annual mean temperature, and annual mean precipitation, for all of the local sampling points, were downloaded from DIVA-GIS 7.5.0 (http://www.diva-gis.org) (Table S2). The ACP laboratory population was reared on orange jessamine (Murraya paniculata) seedlings under controlled conditions at 26°C ± 2°C with 40 to 50% relative humidity and a 14-h/10-h (light/dark) photoperiod over 10 generations. All collected ACP samples were preserved in 100% ethanol and stored at −20°C until the DNA was extracted.

### DNA extraction, amplicon library preparation, and sequencing.

Every biological replicate included four mixed-gender ACP adults and four biological replicates per population. Before DNA extraction, sterile water was used to wash the surface of individual samples three times. The DNeasy blood and tissue kit (Qiagen, Hilden, Germany) was used to extract DNA from the samples according to the manufacturer’s instructions. The DNA extracts were then used to generate amplicons with a PCR-based approach. Briefly, the universal primer pair 806R (5′-GGACTACHVGGGTWTCTAAT-3′) and 338F (5′-ACTCCTACGGGAGGCAGCAG-3′) was used to amplify the bacterial 16S rRNA gene fragments (V3-V4) by PCR. The reaction conditions for PCR included a 20-μL reaction mixture volume containing 0.8 μL of the forward/reverse primer (5 μmol), 0.2 μL of bovine serum albumin, 2 μL of 2.5 mM deoxynucleoside triphosphates (dNTPs), 4 μL of 5× Fast*Pfu* buffer, and 10 ng of DNA. The PCR procedures were performed at 95°C for 3 min, 95°C for 30 s, 55°C for 30 s, and 72°C for 45 s with 27 amplification cycles, with a final extension step at 72°C for 10 min. The PCR products were visualized on 1.2% agarose gels, and the fragments were extracted using a TaKaRa (Nojihigashi, Japan) MiniBEST version 4.0 agarose gel DNA extraction kit. Specific barcodes and Illumina sequencing adapters were added to the purified products, and a TruePrep V3 index kit for Illumina (Vazyme, Nanjing, China) was used to start the second round of PCR. Hieff NGS DNA selection beads (Yeasen, Shanghai, China) were used to purify the final PCR products. They were equalized and normalized using the dsDNA HS (double-stranded DNA high-sensitivity) assay kit for Qubit (Yeasen). A Qubit 4 fluorometer (Invitrogen, Carlsbad, CA, USA) was used to quantify and pool the DNA at an equimolar ratio for all of the samples. The samples were then subjected to high-throughput sequencing on an Illumina (San Diego, CA, USA) MiSeq PE300 (300-bp paired-end) platform by Majorbio Bio-Pharm Technology Co., Ltd., Shanghai, China.

### Bacterial community analyses.

We used Trimmomatic to demultiplex and quality filter the raw FASTQ files and merged the files using FLASH (v.1.2.7) (36). Amplicon sequence variants (ASVs) were clustered at 97% similarity by using QIIME 2 (version 2020.2) (37), and the RDP classifier was applied to phylogenetically assign taxonomic classifications (38) (http://rdp.cme.msu.edu/). The samples were pure to 30,002 sequences (based on the group that had the lowest coverage in all of the samples) to normalize the sequencing depth. Bray-Curtis dissimilarity metrics for all of the samples were used for β-diversity.py in QIIME 2 (37) (http://qiime.sourceforge.net/). They were viewed directly by a principal-coordinate analysis (PCoA) and nonmetric multidimensional scaling (NMDS) (39, 40). Linear discriminant analysis effect size (LEfSe) measurements (41) (http://huttenhower.sph.harvard.edu/galaxy/root?tool_id=lefse_upload) were also conducted. ADONIS (42) was used to statistically assess the differences between microbial communities in different populations. Network analyses were performed to investigate the relationships between different ASVs by the sparse correlations for the compositional data algorithm operation in the stat Python module. Spearman’s correlation coefficients of >0.6 and false discovery rate-corrected *P* values of <0.05 were used to filter the intimate connections (43). To describe the topology of the networks, a set of metrics, including nodes, edges, positive and negative edges, average degrees, average path lengths, and clustering coefficients, was calculated. The visualization was generated using Gephi (44).

### Amplification and sequencing of *Wolbachia* in ACP populations.

To identify the *Wolbachia* strains in different ACP populations, five ACP adults were randomly selected from each population. DNA samples were obtained from each individual. Fragments of five genes (*coxA*, *fbpA*, *ftsZ*, *gatB*, and *hcpA*) of *Wolbachia* were sequenced from all of the samples. These genes were amplified using specific primers and PCR conditions (45, 46) (Table 2). The final concentration of the multilocus sequence typing (MLST) primer pairs was 1 μmol/L, and the final volume was 20 μL for all reaction mixtures. The PCR products were identified and extracted as described above. The products were then submitted to the Beijing Genomics Institute (Beijing, China) for sequencing (from both the forward and reverse ends). To investigate the evolutionary relationships of the *Wolbachia* strains from field-collected ACPs, the nucleotide sequences were aligned using ClustalW with default settings in the MLST database (47). A phylogenetic tree based on constructed in MEGA v7.0 using the neighbor-joining method, and bootstrap values were calculated based on 1,000 replicates (48).

**TABLE 2:** Primers used for *Wolbachia* identification in this study

Target	Primer sequence (5′→3′)	Amplicon size (bp)	Annealing temp (°C)
*gatB*	Forward, GAKTTAAAYCGYGCAGGBGTT	471	54
	Reverse, TGGYAAYTCRGGYAAAGATGA		

*coxA*	Forward, TTGGRGCRATYAACTTTATAG	487	54
	Reverse, CTAAAGACTTTKACRCCAGT		

*hcpA*	Forward, GAAATARCAGTTGCTGCAAA	515	54
	Reverse, GAAAGTYRAGCAAGYTCTG		

*ftsZ*	Forward, ATYATGGARCATATAAARGATAG	524	52
	Reverse, TCRAGYAATGGATTRGATAT		

*fbpA*	Forward, GCTGCTCCRCTTGGYWTGAT	509	58
	Reverse, CCRCCAGARAAAAYYACTATTC		

### Association analyses of environmental variables and bacterial communities.

We used Moran’s *I* (49) to analyze the spatial autocorrelation of the primary bacteria in field-collected ACPs, including *Ca*. Profftella, *Wolbachia*, *Pantoea*, “*Ca.* Liberibacter asiaticus,” *Lactobacillus*, and Acinetobacter. A structural equation model (SEM) (50) with a Satorra-Bentler correction was used to evaluate the effects of geographic or climatic factors on the predominant ACP symbionts *Ca*. Profftella and *Wolbachia*. To exclude errors induced by different deviances in the parameters, a standardized coefficient was introduced to measure the linear relationship of every model path. We selected the simple model with the lowest Akaike information criterion (AIC) value (51). All of the statistical analyses were conducted in R version 4.0.1. The statistical significance of differences in data between different geographical samples was determined using a Newman-Keuls test and Student’s *t* test. Differences were considered to be significant when the *P* values were <0.05. These data analyses were conducted using SPSS 21 (IBM, Inc., Armonk, NY, USA).

### Data availability.

Molecular sequence data have been deposited in the NCBI Sequence Read Archive (SRA) database (BioProject accession number PRJNA912851).
