# Supplementary material for: Environmental Factors Affect the Bacterial Community in Diaphorina citri, an Important Vector of “Candidatus Liberibacter asiaticus”
Source: Microbiol Spectr. 2023 Mar 28;11(2):e05298-22. doi: 10.1128/spectrum.05298-22 (PMC10100744; doi:10.1128/spectrum.05298-22)
Supplement: Supplemental file 1 — Supplemental material. Download spectrum.05298-22-s0001.pdf, PDF file, 1.0 MB [file spectrum.05298-22-s0001.pdf]

Supplementary materials

Environmental factors affect the bacterial community in *Diaphorina citri*, an important vector of *Candidatus* Liberibacter asiaticus

Rui-Xu Jiang [a,b], Feng Shang [a,b], Hong-Bo Jiang [a,b], Wei Dou [a,b], Tomislav Cernava [c], Jin-Jun Wang[a,b*]

[a] Key Laboratory of Entomology and Pest Control Engineering, College of Plant Protection, Southwest University, Chongqing, China.

[b] International Joint Laboratory of China-Belgium on Sustainable Crop Pest Control, Academy of Agricultural Sciences, Southwest University, Chongqing, China

[c] Institute of Environmental Biotechnology, Graz University of Technology, Graz, Austria

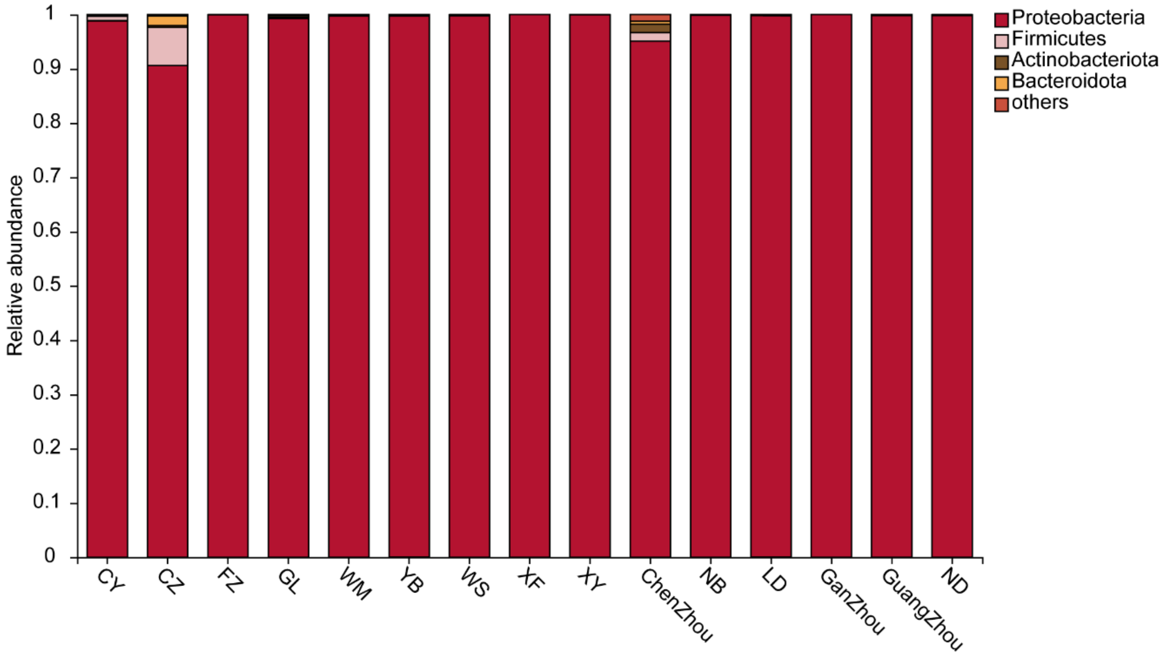

Fig. S1. Community structure of the bacterial community in different field ACP populations at the phylum level. ACP, Asian citrus psyllid.

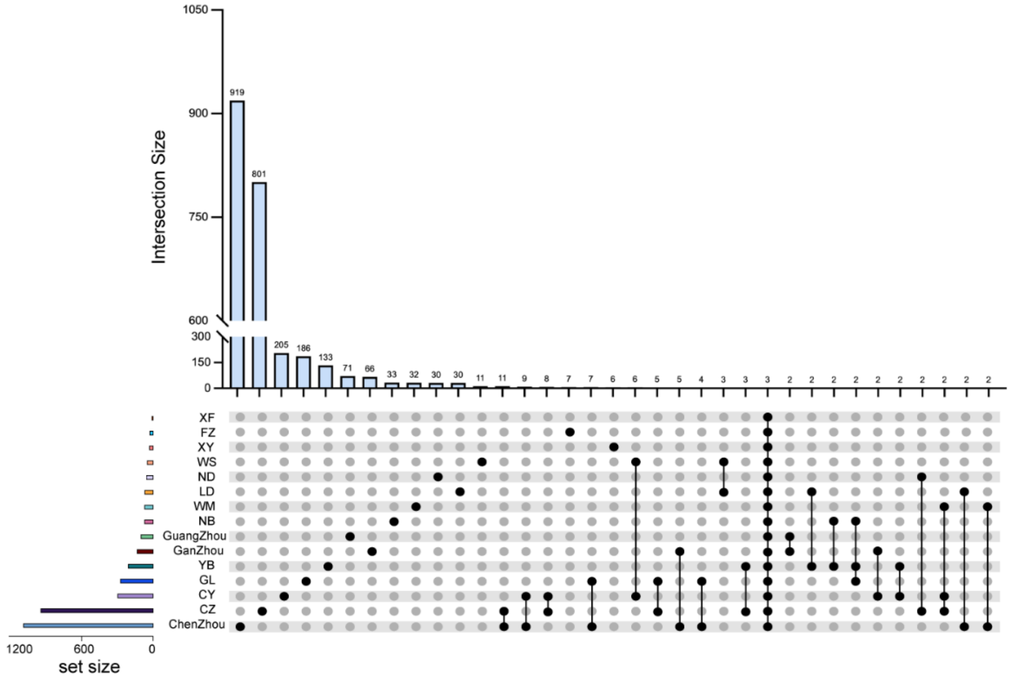

Fig. S2. Setup diagram of shared and unique ASVs numbers observed in the different field ACP populations. ACP, Asian citrus psyllid; ASV, amplicon sequence variant.

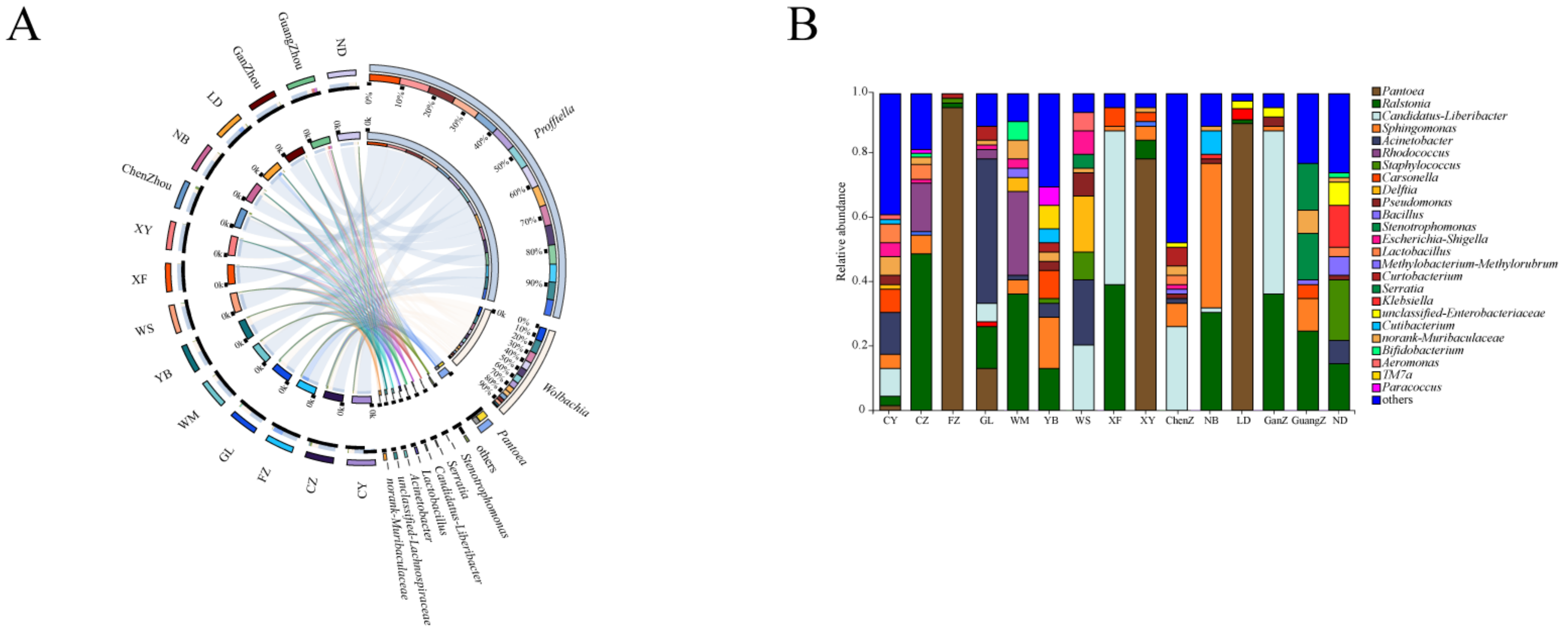

Fig. S3. Bacterial community structure from the different populations of ACP. (A) The bacterial proportion in all the ACP populations. (B) Exclusion of *Profftella* and *Wolbachia* as dominant bacteria in the ACP populations. Other genera ("others") account for < 5% of the classified sequences. ACP, Asian citrus psyllid.

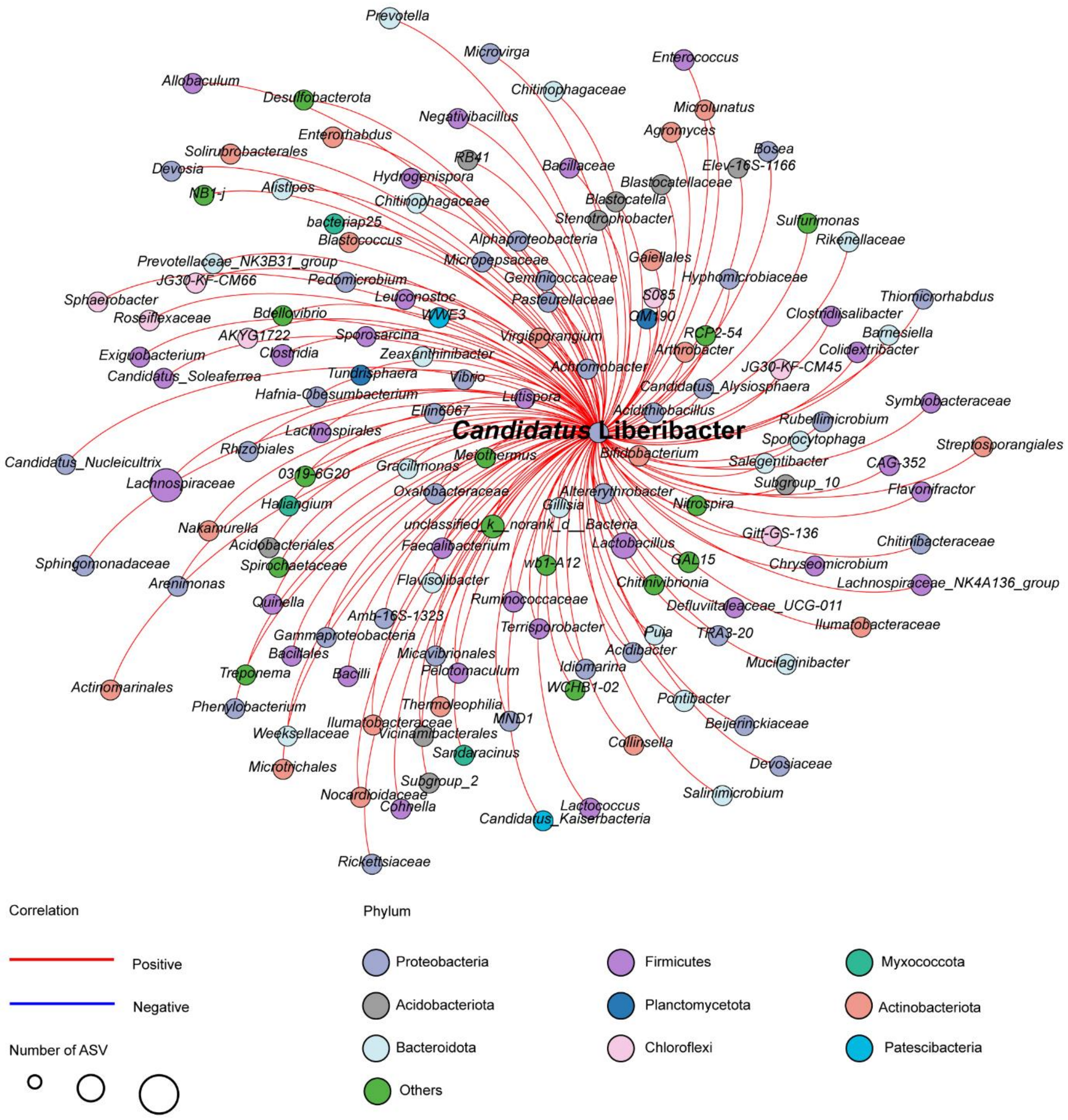

Fig. S4. Network of bacterial communities in the *Candidatus* Liberibacter asiaticus with bacteria in the field population of ACP adult samples. ACP, Asian citrus psyllid.

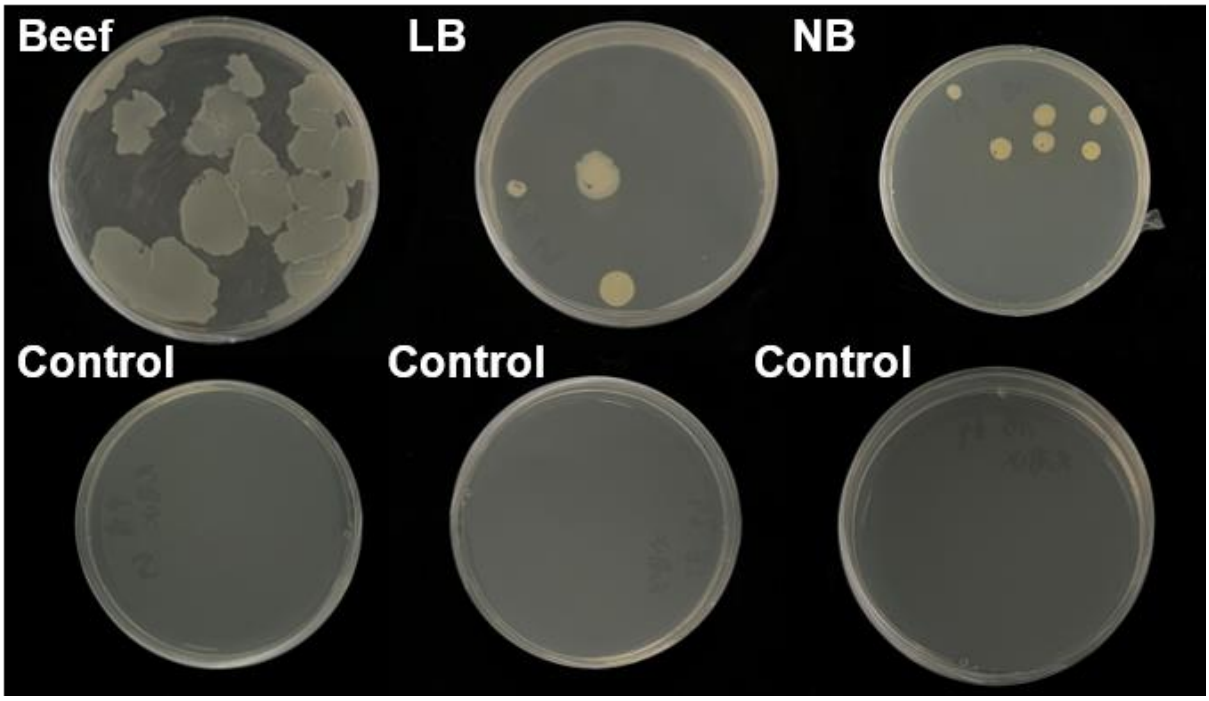

Fig. S5. Isolation of ACP-associated bacteria on different cultivation media. ACP, Asian citrus psyllid.

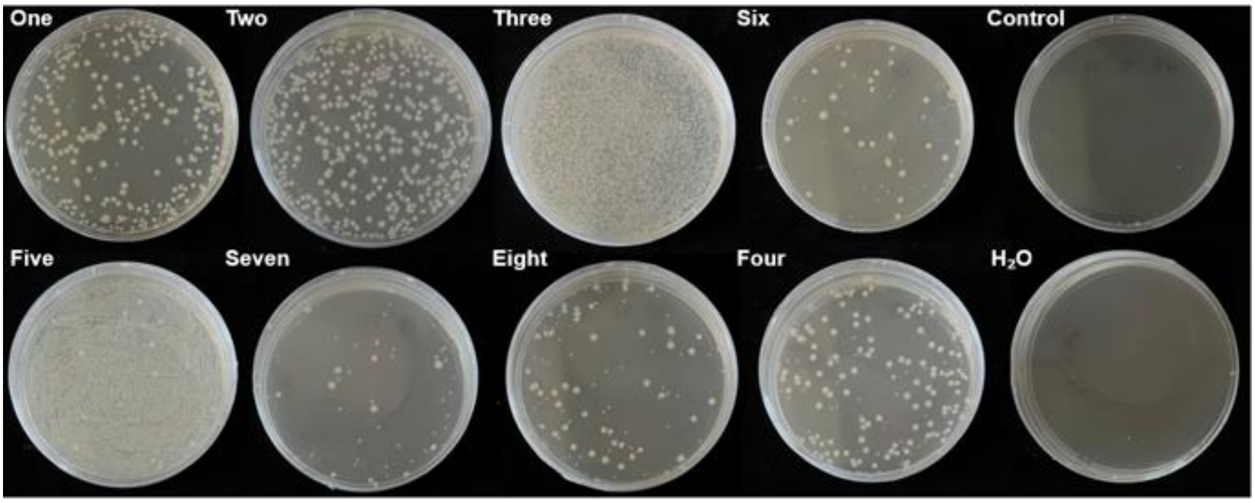

Fig. S6. Isolation of bacteria associated with ACP on different cultivation media. ACP, Asian citrus psyllid. Isolation of bacteria associated with ACP with different methods and the same medium. ACP, Asian citrus psyllid.

Table S1 Summary of the collection details. The population code (ID), province, city, county, latitude, longitude, and collection date

ID	Province	City/Country	Host	Latitude	Longitude	Altitude	Date
Ganzhou (GanZhou)	Jiangxi	Ganzhou	Newhall	25.78°N	114.52°E	189	04/30/2021
Chongyi (CY)	Jiangxi	Chongyi	Newhall	25.65°N	114.28°E	430	07/16/2021
Xinfeng (XF)	Jiangxi	Xinfeng	Newhall	25.48°N	114.95°E	205	09/06/2021
Xinyu (XY)	Jiangxi	Xinyu	(Tangerine) Miju	27.81°N	114.59°E	80	09/08/2021
Fuzhou (FZ)	Jiangxi	Fuzhou	(Tangerine) Miju	27.55°N	116.59°E	100	09/28/2021
Guilin (GL)	Guangxi	Guilin	Shatangju	25.44°N	110.28°E	177	03/26/2021
Chongzuo (CZ)	Guangxi	Chongzuo	(Orah) Wogan	22.36°N	107.40°E	155	03/22/2021
Wuming (WM)	Guangxi	Wuming	(Orah) Wogan	23.02°N	108.34°E	168	03/24/2021
Ningbo (NB)	Zhejiang	Ningbo	(Owari satsuma)Dayeweizhang	29.34°N	121.90°E	118	09/27/2021
Ningde (ND)	Fujian	Ningde	(Hybrid Citrus) Zagan	26.48°N	118.67°E	565	04/25/2021
Luodian (LD)	Guizhou	Luodian	Newhall	25.48°N	106.76°E	573	06/10/2021
Wenshan (WS)	Yunnan	Wenshan	(Hybrid Citrus) Zagan	23.10°N	104.51°E	1002	06/15/2021
Chenzhou (ChenZhou)	Hunan	Chenzhou	Shatangju	25.40°N	112.90°E	634	08/15/2021
Guangzhou (GuangZhou)	Guangdong	Guangzhou	Shatangju	23.18°N	113.38°E	56	09/04/2021
Yibin (YB)	Sichuan	Yibing Anbian	(Hybrid Citrus) Zagan	28.62°N	104.44°E	461	09/13/2021

Table S2 Annual mean temperatures (AMT) and the annual mean precipitation (AMP) of the 15 locations obtained from DIVA-GIS 7.5.0

ID	AMT	AMP
Ganzhou (GanZhou)	21.05	1508
Chongyi (CY)	19.9	1589
Xinfeng (XF)	21.6	1526
Xinyu (XY)	20.2	1421
Fuzhou (FZ)	20.05	1673
Guilin (GL)	20.9	1643
Chongzuo (CZ)	24.1	1810
Wuming (WM)	23.6	1713
Ningbo (NB)	18.6	1773
Ningde (ND)	19.7	1747
Luodian (LD)	21.4	1374
Wenshan (WS)	20.8	1803
Chenzhou (ChenZhou)	20.3	1429
Guangzhou (GuangZhou)	23.6	1955
Yibin(YB)	19.3	1243

Table S3 Measures of species richness and diversity of 15 field ACP populations

Sample	Sobs	Ace	Chao	Shannon	Simpson	Coverage
Chongyi (CY)	89	93.737	91.321	0.770	0.640	0.999
Chongzuo (CZ)	16	16.45	16.250	0.687	0.599	0.999
Fuzhou (FZ)	12	15.478	12.375	0.652	0.649	0.999
Ningde (ND)	18	19.184	18.800	0.658	0.613	0.999
Guangzhou (GuangZhou)	28	32.308	31.220	0.928	0.523	0.997
Guilin (GL)	46	48.813	48.795	1.274	0.361	0.997
Ganzhou (GanZhou)	9	9.672	9.500	0.414	0.778	0.999
Luodian (LD)	25	25.433	25.050	1.073	0.466	0.999
Xinyu (XY)	9.75	10.84	10.040	0.165	0.934	0.999
Ningbo (NB)	22	25.677	26.071	0.827	0.514	0.997
Chenzhou (ChenZhou)	284	306.215	298.200	1.113	0.604	0.997
Wuming (WM)	23	23.141	22.778	0.767	0.555	0.999
Wenshan (WS)	18	19.066	18.546	0.576	0.703	0.999
Xinfeng (XF)	6	6.609	6.250	0.111	0.959	0.999
Yibin(YB)	34	46.825	37.949	0.945	0.453	0.999

Note: ACP, Asian citrus psyllid

Table S4 The spatial autocorrelation estimated by Moran’s I of different parameters

Parameters	Moran’s I		Sd	*P*
	Observed value	Expected value		
Altitude (ALT)	-0.118	-0.017	0.012	<.001
Annual mean temperature (Bio1)	-0.165	-0.017	0.012	<.001
Annual precipitation (Bio12)	-0.072	-0.017	0.012	<.001
Profftella proportion	-0.043	-0.017	0.012	0.024
Wolbachia proportion	-0.081	-0.017	0.012	<.001
Pantoea proportion	-0.022	-0.017	0.010	0.587
*C*Las proportion	-0.019	-0.017	0.010	0.845
Lactobacillus proportion	-0.039	-0.017	0.010	0.032
Actinetobacter proportion	-0.049	-0.017	0.010	0.002

Table S5 Total effects on the proportion of *Wolbachia* and *Profftella* estimated by the SEM model

Effects	*Wolbachia* proportion			*Profftella* proportion
	Coefficient ± SE	*z*	*P*	Coefficient ± SE
Direct effects of spatial factors	-1.41±0.29	4.9	<.001	-0.14±0.11
Indirect effects of climate factors	1.40±0.19	7.31	<.001	0.022±0.074
Total effects	-0.0020±0.18	0.013	0.99	-0.12±0.14

Note: SEM, structural equation model.

Table S6 Topological property of bacterial networks of ACP bacterial relationship with *Candidatus* Liberibacter asiaticus

Source	Target	Correlation	*P* value	Positive/ Negative
Ellin6067	*Candidatus* Liberibacter	0.799957	<0.000001	Positive
Idiomarina	*Candidatus* Liberibacter	0.799957	<0.000001	Positive
Enterorhabdus	*Candidatus* Liberibacter	0.796268	<0.000001	Positive
Stenotrophobacter	*Candidatus* Liberibacter	0.793172	<0.000001	Positive
Salegentibacter	*Candidatus* Liberibacter	0.792732	<0.000001	Positive
Micavibrionales	*Candidatus* Liberibacter	0.786723	<0.000001	Positive
Subgroup_10	*Candidatus* Liberibacter	0.783088	<0.000001	Positive
Enterococcus	*Candidatus* Liberibacter	0.782715	<0.000001	Positive
Bosea	*Candidatus* Liberibacter	0.779579	<0.000001	Positive
Symbiobacteraceae	*Candidatus* Liberibacter	0.779579	<0.000001	Positive
Sporosarcina	*Candidatus* Liberibacter	0.779579	<0.000001	Positive
Thermoleophilia	*Candidatus* Liberibacter	0.779579	<0.000001	Positive
Sporocytophaga	*Candidatus* Liberibacter	0.779579	<0.000001	Positive
Nakamurella	*Candidatus* Liberibacter	0.779579	<0.000001	Positive
Haliangium	*Candidatus* Liberibacter	0.779579	<0.000001	Positive
Hyphomicrobiaceae	*Candidatus* Liberibacter	0.779579	<0.000001	Positive
Rickettsiaceae	*Candidatus* Liberibacter	0.779579	<0.000001	Positive
Candidatus_Alysiosphaera	*Candidatus* Liberibacter	0.779579	<0.000001	Positive
Achromobacter	*Candidatus* Liberibacter	0.779579	<0.000001	Positive
Spirochaetaceae	*Candidatus* Liberibacter	0.779579	<0.000001	Positive
Vibrio	*Candidatus* Liberibacter	0.779579	<0.000001	Positive
Chitinophagaceae	*Candidatus* Liberibacter	0.779579	<0.000001	Positive
Virgisporangium	*Candidatus* Liberibacter	0.779579	<0.000001	Positive
wb1-A12	*Candidatus* Liberibacter	0.779579	<0.000001	Positive
Acidithiobacillus	*Candidatus* Liberibacter	0.779579	<0.000001	Positive
TRA3-20	*Candidatus* Liberibacter	0.779579	<0.000001	Positive
Ilumatobacteraceae	*Candidatus* Liberibacter	0.779579	<0.000001	Positive
Sandaracinus	*Candidatus* Liberibacter	0.779579	<0.000001	Positive
Lutispora	*Candidatus* Liberibacter	0.779579	<0.000001	Positive
Quinella	*Candidatus* Liberibacter	0.779579	<0.000001	Positive
Gracilimonas	*Candidatus* Liberibacter	0.779579	<0.000001	Positive
Ilumatobacteraceae	*Candidatus* Liberibacter	0.779579	<0.000001	Positive
Salinimicrobium	*Candidatus* Liberibacter	0.779579	<0.000001	Positive

Candidatus_Nucleicultrix	*Candidatus* Liberibacter	0.779579	<0.000001	Positive
Sphaerobacter	*Candidatus* Liberibacter	0.779579	<0.000001	Positive
Puia	*Candidatus* Liberibacter	0.779579	<0.000001	Positive
WWE3	*Candidatus* Liberibacter	0.779579	<0.000001	Positive
Clostridiisalibacter	*Candidatus* Liberibacter	0.779579	<0.000001	Positive
Chryseomicrobium	*Candidatus* Liberibacter	0.779579	<0.000001	Positive
CAG-352	*Candidatus* Liberibacter	0.779579	<0.000001	Positive
OM190	*Candidatus* Liberibacter	0.779579	<0.000001	Positive
JG30-KF-CM66	*Candidatus* Liberibacter	0.779579	<0.000001	Positive
Flavonifractor	*Candidatus* Liberibacter	0.779579	<0.000001	Positive
WCHB1-02	*Candidatus* Liberibacter	0.779579	<0.000001	Positive
Negativibacillus	*Candidatus* Liberibacter	0.779579	<0.000001	Positive
Devosia	*Candidatus* Liberibacter	0.779579	<0.000001	Positive
Chitinibacteraceae	*Candidatus* Liberibacter	0.779579	<0.000001	Positive
Pelotomaculum	*Candidatus* Liberibacter	0.779579	<0.000001	Positive
Chitinophagaceae	*Candidatus* Liberibacter	0.779579	<0.000001	Positive
Chitinivibrionia	*Candidatus* Liberibacter	0.779579	<0.000001	Positive
Zeaxanthinibacter	*Candidatus* Liberibacter	0.779579	<0.000001	Positive
Lachnospirales	*Candidatus* Liberibacter	0.779579	<0.000001	Positive
Bacillaceae	*Candidatus* Liberibacter	0.779579	<0.000001	Positive
Defluviitaleaceae_UCG-011	*Candidatus* Liberibacter	0.779579	<0.000001	Positive
Microvirga	*Candidatus* Liberibacter	0.779579	<0.000001	Positive
Hydrogenispora	*Candidatus* Liberibacter	0.779579	<0.000001	Positive
Bacillales	*Candidatus* Liberibacter	0.779579	<0.000001	Positive
Geminicoccaceae	*Candidatus* Liberibacter	0.779579	<0.000001	Positive
RB41	*Candidatus* Liberibacter	0.779579	<0.000001	Positive
Sulfurimonas	*Candidatus* Liberibacter	0.779579	<0.000001	Positive
Acidobacteriales	*Candidatus* Liberibacter	0.779579	<0.000001	Positive
Thiomicrorhabdus	*Candidatus* Liberibacter	0.779579	<0.000001	Positive
Exiguobacterium	*Candidatus* Liberibacter	0.779579	<0.000001	Positive
Devosiaceae	*Candidatus* Liberibacter	0.779579	<0.000001	Positive
Elev-16S-1166	*Candidatus* Liberibacter	0.779579	<0.000001	Positive
Barnesiella	*Candidatus* Liberibacter	0.779579	<0.000001	Positive
Rubellimicrobium	*Candidatus* Liberibacter	0.779579	<0.000001	Positive
Leuconostoc	*Candidatus* Liberibacter	0.779579	<0.000001	Positive

Candidatus_Soleaferrea	*Candidatus* Liberibacter	0.779579	<0.000001	Positive
bacteriap25	*Candidatus* Liberibacter	0.779579	<0.000001	Positive
Blastococcus	*Candidatus* Liberibacter	0.779579	<0.000001	Positive
Streptosporangiales	*Candidatus* Liberibacter	0.779579	<0.000001	Positive
Weeksellaceae	*Candidatus* Liberibacter	0.779579	<0.000001	Positive
Cohnella	*Candidatus* Liberibacter	0.779579	<0.000001	Positive
Microlunatus	*Candidatus* Liberibacter	0.779579	<0.000001	Positive
Mucilaginibacter	*Candidatus* Liberibacter	0.773345	<0.000001	Positive
Micropepsaceae	*Candidatus* Liberibacter	0.765002	<0.000001	Positive
MND1	*Candidatus* Liberibacter	0.761923	<0.000001	Positive
Rhizobiales	*Candidatus* Liberibacter	0.759983	<0.000001	Positive
Prevotellaceae_NK3B31_group	*Candidatus* Liberibacter	0.754561	<0.000001	Positive
Gitt-GS-136	*Candidatus* Liberibacter	0.750756	<0.000001	Positive
Arenimonas	*Candidatus* Liberibacter	0.750756	<0.000001	Positive
Bifidobacterium	*Candidatus* Liberibacter	0.75014	<0.000001	Positive
Amb-16S-1323	*Candidatus* Liberibacter	0.745441	<0.000001	Positive
Prevotella	*Candidatus* Liberibacter	0.740012	<0.000001	Positive
Allobaculum	*Candidatus* Liberibacter	0.736739	0.000001	Positive
Vicinamibacterales	*Candidatus* Liberibacter	0.720792	0.000001	Positive
GAL15	*Candidatus* Liberibacter	0.714872	0.000002	Positive
Actinomarinales	*Candidatus* Liberibacter	0.711794	0.000002	Positive
Lactococcus	*Candidatus* Liberibacter	0.711157	0.000003	Positive
Roseiflexaceae	*Candidatus* Liberibacter	0.704941	0.000003	Positive
Microtrichales	*Candidatus* Liberibacter	0.700749	0.000004	Positive
Subgroup_2	*Candidatus* Liberibacter	0.70063	0.000004	Positive
Pasteurellaceae	*Candidatus* Liberibacter	0.696743	0.000005	Positive
Gillisia	*Candidatus* Liberibacter	0.688791	0.000008	Positive
Bacilli	*Candidatus* Liberibacter	0.684051	0.00001	Positive
Lachnospiraceae_NK4A136_group	*Candidatus* Liberibacter	0.683558	0.00001	Positive
Pontibacter	*Candidatus* Liberibacter	0.678101	0.000013	Positive
Nitrospira	*Candidatus* Liberibacter	0.67679	0.000013	Positive
Ruminococcaceae	*Candidatus* Liberibacter	0.67644	0.000013	Positive
0319-6G20	*Candidatus* Liberibacter	0.67145	0.000017	Positive
Blastocatellaceae	*Candidatus* Liberibacter	0.670926	0.000017	Positive
S085	*Candidatus* Liberibacter	0.666537	0.000021	Positive

AKYG1722	*Candidatus* Liberibacter	0.664102	0.000023	Positive
Colidextribacter	*Candidatus* Liberibacter	0.663826	0.000023	Positive
Rikenellaceae	*Candidatus* Liberibacter	0.663826	0.000023	Positive
Treponema	*Candidatus* Liberibacter	0.663826	0.000023	Positive
Agromyces	*Candidatus* Liberibacter	0.663826	0.000023	Positive
Gammaproteobacteria	*Candidatus* Liberibacter	0.642283	0.000055	Positive
Nocardioidaceae	*Candidatus* Liberibacter	0.639066	0.000062	Positive
Faecalibacterium	*Candidatus* Liberibacter	0.634599	0.000074	Positive
Gaiellales	*Candidatus* Liberibacter	0.634526	0.000074	Positive
Desulfobacterota	*Candidatus* Liberibacter	0.632321	0.00008	Positive
Pedomicrobium	*Candidatus* Liberibacter	0.629001	0.000091	Positive
Hafnia-Obesumbacterium	*Candidatus* Liberibacter	0.626641	0.000099	Positive
Bdellovibrio	*Candidatus* Liberibacter	0.61808	0.000134	Positive
Altererythrobacter	*Candidatus* Liberibacter	0.616529	0.000141	Positive
Collinsella	*Candidatus* Liberibacter	0.612114	0.000164	Positive
Solirubrobacterales	*Candidatus* Liberibacter	0.61162	0.000167	Positive
Alistipes	*Candidatus* Liberibacter	0.608952	0.000182	Positive
Blastocatella	*Candidatus* Liberibacter	0.606497	0.000198	Positive
Meiothermus	*Candidatus* Liberibacter	0.605373	0.000205	Positive
NB1-j	*Candidatus* Liberibacter	0.605373	0.000205	Positive
Acidibacter	*Candidatus* Liberibacter	0.590868	0.000327	Positive
Terrisporobacter	*Candidatus* Liberibacter	0.584213	0.000401	Positive
Arthrobacter	*Candidatus* Liberibacter	0.575928	0.000515	Positive
Alphaproteobacteria	*Candidatus* Liberibacter	0.575928	0.000515	Positive
unclassified_k__norank_d__Bacteria	*Candidatus* Liberibacter	0.574903	0.00053	Positive
Oxalobacteraceae	*Candidatus* Liberibacter	0.571159	0.000592	Positive
Beijerinckiaceae	*Candidatus* Liberibacter	0.55935	0.000827	Positive
Flavisolibacter	*Candidatus* Liberibacter	0.5579	0.000861	Positive
Sphingomonadaceae	*Candidatus* Liberibacter	0.549443	0.001083	Positive
Phenylobacterium	*Candidatus* Liberibacter	0.542651	0.001297	Positive
JG30-KF-CM45	*Candidatus* Liberibacter	0.529315	0.001823	Positive
Tundrisphaera	*Candidatus* Liberibacter	0.524666	0.002045	Positive
Lachnospiraceae	*Candidatus* Liberibacter	0.523359	0.002111	Positive
RCP2-54	*Candidatus* Liberibacter	0.523216	0.002119	Positive
Lactobacillus	*Candidatus* Liberibacter	0.518963	0.002349	Positive

Candidatus_Kaiserbacteria	*Candidatus* Liberibacter	0.511496	0.002804	Positive
Clostridia	*Candidatus* Liberibacter	0.500633	0.003601	Positive

Note: ACP, Asian citrus psyllid

Table S7 Bacterial co-occurrence network characteristics in field and laboratory population

Group	Nodes	Edges	Positive edges	Negative edges	Avg. degree	Avg. path length	Clustering coefficient
Field	58	308	304	4	10.62	0	0.97
Laboratory	92	2522	2521	1	54.83	0	1
